# Effects of campaign for postpartum vaccination on seronegative rate against rubella among Japanese women

**DOI:** 10.1186/1471-2334-14-152

**Published:** 2014-03-21

**Authors:** Takahiro Yamada, Junko Mochizuki, Masachi Hanaoka, Eriko Hashimoto, Akihide Ohkuchi, Mika Ito, Takahiko Kubo, Akihito Nakai, Shigeru Saito, Nobuya Unno, Shigeki Matsubara, Hisanori Minakami

**Affiliations:** 1 Department of Obstetrics, Hokkaido University Graduate School of Medicine, N15W7, Kita-ku, Sapporo 060-8638, Japan; 2 Department of Obstetric and Gynecology, School of Medicine, Kitasato University, Sagamihara, Japan; 3 Department of Maternal-Fetal and Neonatal Medicine, National Center for Child Health and Development, Tokyo, Japan; 4 Department of Obstetric and Gynecology, Nippon Medical School, Tokyo, Japan; 5 Department of Obstetric and Gynecology, Jichi Medical University School of Medicine, Shimotsuke, Japan; 6 Graduate School of Medicine and Pharmaceutical Science, University of Toyama, Sugitani, Japan

**Keywords:** Congenital rubella syndrome, Outbreak, Vaccine

## Abstract

**Background:**

Japan experienced two rubella outbreaks in the past decade (2004 and 2012 – 2013), resulting in 10 and 20 infants with congenital rubella syndrome (CRS), respectively. This study was performed to determine whether the seronegative rate was lower in multiparous women than in primiparous women in Japan.

**Methods:**

Hemagglutination inhibition (HI) test results during pregnancy were analyzed retrospectively in 11048 primiparous and 9315 multiparous women who gave birth at six hospitals in northern Japan in the 5-year study period (January 2008 through December 2012). Women with HI titer <  1:8 were defined as susceptible to rubella.

**Results:**

The seronegative rate was significantly lower in multiparous than primiparous women aged 30 – 31 years (2.3% [22/967] vs. 4.5% [66/1454], *P*  =  0.0036), 36 – 37 years (3.4% [55/1601] vs. 5.7% [79/1389], *P*  =  0.0030), and overall women (3.8% [350/9315] aged 34.7  ±  5.2 vs. 5.4% [597/11048] for 33.2  ±  5.9, *P*  <  0.001). The susceptible fraction size did not differ largely according to hospital, ranging from 3.5% to 6.3%. Those for each year did not change markedly; 4.5% [150/3369], 5.2% [221/4268], 4.4% [195/4412], 4.6% [186/4056], and 4.6% [195/4258] for 2008, 2009, 2010, 2011, and 2012, respectively. Those for teenagers were consistently high: 22.7% [5/22], 20.7% [6/29], 20.6% [7/34], 13.0% [3/23], and 23.5% [4/17] for 2008, 2009, 2010, 2011, and 2012, respectively.

**Conclusions:**

The seronegative rate was significantly lower in multiparous than primiparous women. However, Japanese rubella vaccination programs were insufficient to eliminate CRS.

## Background

Public health concern regarding rubella stems from the teratogenic effects that can result from congenital rubella infection, particularly during the first trimester of pregnancy. Japan experienced a rubella outbreak in 2004, in which 10 infants contracted congenital rubella syndrome (CRS) [1]. Supplemental immunization activity targeting adult women and population immunity surveys were strengthened since the outbreak in 2004. Japanese guidelines for obstetric practice recommend determination of immunity status against rubella with hemagglutination inhibition (HI) test during the first trimester and postpartum vaccination in women with low titer of HI test results (≤ 16×) [2].

However, a rubella outbreak occurred again in Japan in 2012 – 2013 [3]. The total number of rubella patients in Japan during the first 9 months of 2013 was 14077 (108 per 1000000 population, 69% of cases were serologically confirmed) [3]. Among 14077 patients in this outbreak, vaccination status was unknown in 8973 patients. Of 5104 patients with known vaccination status, 924 (18.1%) had been vaccinated, while 4180 (81.9%) had not been vaccinated [4]. The majority of rubella cases occurred among adults aged 18 years or older: male and female adults aged 18 years or older accounted for 71.7% and 19.8% of all 14077 cases, respectively [3]. Consequently, 20 infants (1.8 per 100000 live births) were diagnosed with CRS during the 12-month period between October 2012 and September 2013 in Japan [3].

The present retrospective and multicenter study was conducted to determine whether the experience of prior birth influenced seronegative rate against rubella among pregnant Japanese women and to assess how many pregnant Japanese women were susceptible to rubella during the rubella outbreak that occurred in Japan in 2012 – 2013.

## Methods

This study was conducted after being approved by the Institutional Review Boards of Hokkaido University Hospital, Kitasato University Hospital, National Center for Child Health and Development, Nippon Medical School Tama-Nagayama Hospital, Jichi Medical University Hospital, and Toyama University Hospital.

This retrospective study included 20363 women, all of whom fulfilled the following criteria: rubella immunity was determined in pregnancy by HI test and gave birth during the 5-year period between January 2008 and December 2012 at one of following six hospitals located in northern Japan: Hokkaido University Hospital, Toyama University Hospital, Jichi Medical University Hospital, National Center for Child Health and Development, Nippon Medical School Nagayama Hospital, and Kitasato University Hospital (Table 1). A portion of the results obtained in this study regarding the overall fraction of pregnant women susceptible to rubella was described elsewhere previously [3].

**Table 1:** Regions (prefectures) and numbers of pregnant women tested

**Regions (prefectures)**	**Direction from Tokyo**	**No. of women**
A (Hokkaido)	830 km N	1450
B (Toyama)	260 km NW	803
C (Tochigi)	100 km N	2467
D (Tokyo 1)	15 km W*	7329
E (Tokyo 2)	40 km W*	3642
F (Kanagawa)	35 km W*	4672

N, north; NW, northwest; W, west; *, from Japan Railroad Tokyo Station.

Titer of rubella antibody determined with HI test was expressed as <  8×, 8×, 16×, 32×, 64×, 128×, 256×, 512×, 1024×, and >  1024×. Women with HI titer <  8× were defined as having no immunity against rubella (susceptible to rubella) in this study. The correlation between HI titer (×, [Log2]) and titer (y, IU/mL [Log2]) determined by enzyme immunoassay (Siemens Healthcare Japan, Tokyo, Japan) is as a follows [5]: y  =  0.736×  +  1.6377.

All data are presented as means  ±  SD. For statistical analysis of categorical data, Fisher’s exact test was applied. The statistical software package StatView 5.0 for Macintosh (SAS Institute Inc. Cary, NC) was used for all data analyses. In all analyses, *P*  <  0.05 was taken to indicate statistical significance.

## Results

### Regional differences in rubella immunity among pregnant women

**Figure 1:**
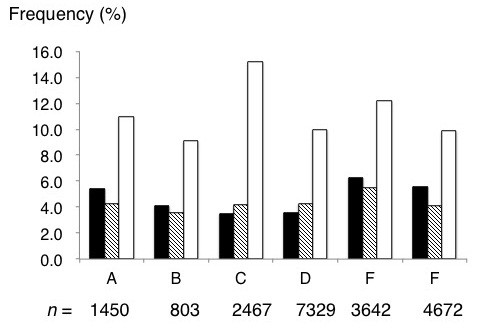
**Regional differences in prevalence rate of pregnant women susceptible to rubella.** A, Hokkaido prefecture; B, Toyama prefecture; C, Tochigi prefecture, D, Tokyo 1; E, Tokyo 2; and F, Kanagawa prefecture. Closed, shaded and open bars indicate hemagglutination inhibition assay (HI) titers of <  8×, 8×, and 16×, respectively. Total number of pregnant women tested is indicated at the bottom.

Regional differences in size of the fraction susceptible to rubella (HI titer <  8×) were relatively small: 3.5% (86/2467) in Region C (Tochigi), 3.5% (257/7329) in Region D (Tokyo 1), 4.1% (33/803) in Region B (Toyama), 5.4% (78/1450) in Region A (Hokkaido), 5.6% (263/4672) in Region F (Kanagawa), and 6.3% (230/3642) in Region E (Tokyo 2) (Figure 1). Overall, 4.7% (947/20363) of all pregnant women were susceptible to rubella. Overall fractions with HI titer 8×, 16×, 32×, 64×, 128×, 256×, 512×, and ≥  1024× were 4.5% (908/20363), 11.0% (2250/20363), 21.5% (4381/20363), 25.5% (5201/20363), 18.7% (3800/20363), 10.1% (2063/20363), 3.5% (715/20363), and 0.5% (98/20363), respectively.

### Fraction of pregnant women susceptible to rubella according to maternal age and year

**Figure 2:**
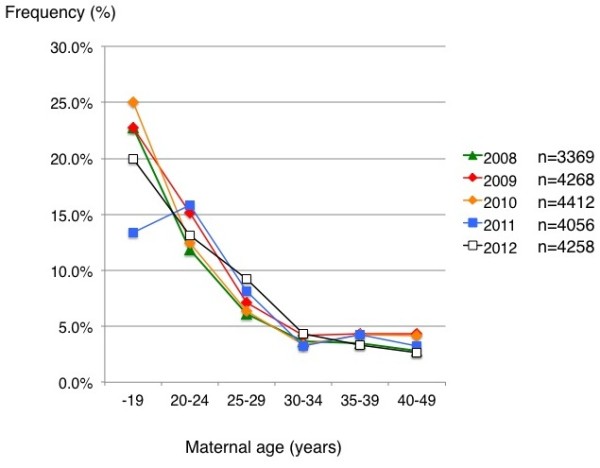
Prevalence rate of pregnant women susceptible to rubella (HI titer <  8×) according to maternal age and year.

The seronegative rates differed greatly between six groups divided according to maternal age (Figure 2). Those for younger women were consistently high: 22.7%[5/22], 20.7%[6/29], 20.6%[7/34], 13.0%[3/23], and 23.5%[4/17] for teenagers, and 11.9%[17/143], 14.3%[28/196], 10.7%[18/168], 13.5%[19/141], and 12.1%[17/140] for women aged 20 – 24 years in 2008, 2009, 2010, 2011, and 2012, respectively. These values decreased with advancing maternal age, and this trend did not vary with time. Overall seronegative rates according to maternal age were as follows [3]: 19.8% [25/126] for women aged ≤  19, 12.6% [99/787] for those aged 20 – 24 years, 7.0% [233/3313] for those aged 25 – 29 years, 3.6% [244/6871] for those aged 30 – 34 years, 3.8% [265/6966] for those aged 35 – 39 years, and 3.5% [81/2300] for those aged ≥  40 years. Overall seronegative rates according to year did not change greatly: 4.5%[150/3369], 5.2%[221/4268], 4.4%[195/4412], 4.6%[186/4056], and 4.6%[195/4258] for 2008, 2009, 2010, 2011, and 2012, respectively.

### Effects of a history of prior birth on the prevalence rate of susceptible pregnant women

Overall, the prevalence rate of susceptibility to rubella was significantly lower in multiparous than in primiparous women (Table 2). However, statistically significant differences in seronegative rate were seen in only a limited number of areas. Furthermore, significant differences in seronegative rates were seen in a limited number of groups of women stratified by age: women aged 30 – 31 years (2.3% [22/967] for multiparous women vs. 4.5% [66/1454] for primiparous women) and women aged 36 – 37 years (3.4% [55/1601] for multiparous women vs. 5.7% [79/1389] for primiparous women) (Table 3).

**Table 2:** Effects of a history of prior birth on frequency of susceptible women to rubella according to area

	**Primiparous**		**Multiparous**	
**Area**	**Age (years)**	**% (a/b)†**	**Age (years)**	**% (a/b)†**
A	30.9 ± 5.3	7.0 (57/816)	32.4 ± 4.9	3.3 (21/634)*
B	28.2 ± 4.8	4.8 (20/416)	34.3 ± 4.3*	3.4 (13/387)
C	30.4 ± 7.7	3.7 (46/1252)	33.0 ± 6.6	3.3 (40/1215)
D	33.2 ± 5.9	4.3 (181/4241)	34.2 ± 5.7	2.5 (76/3088)*
E	29.5 ± 5.6	6.4 (132/2070)	33.4 ± 6.4*	6.2 (98/1572)
F	30.2 ± 7.5	7.1 (161/2253)	32.8 ± 6.7*	4.2 (102/2419)*
Overall	33.2 ± 5.9	5.4 (597/11048)	34.7 ± 5.2*	3.8 (350/9315)*

(a/b)†, number of women with HI titer <  8×/total number of women; *, *P*  <  0.05 vs. corresponding counterpart.

**Table 3:** Effects of a history of prior birth on frequency of susceptible women to rubella according to maternal age

	**No immunity against rubella (HI < 8×)**			
**Age (year)**	**Overall**	**Primiparous**	**Multiparous**	***P*-value**
≤ 19	25/126 (19.8%)	23/111 (20.7%)	2/15 (13.3%)	0.7335
20 – 21	29/184 (15.8%)	23/141 (16.3%)	6/43 (14.0%)	0.7102
22 – 23	43/335 (12.8%)	30/236 (12.7%)	13/99 (13.1%)	0.9166
24 – 25	57/642 (8.9%)	37/451 (8.2%)	20/191 (10.5%)	0.3558
26 – 27	101/1171 (8.6%)	72/816 (8.8%)	29/355 (8.2%)	0.7138
28 – 29	100/1768 (5.7%)	69/1132 (6.1%)	31/636 (5.0%)	0.3549
30 – 31	88/2421 (3.6%)	66/1454 (4.5%)	22/967 (2.3%)	0.0036
32 – 33	103/2881 (3.6%)	60/1484 (4.0%)	43/1397 (3.1%)	0.1632
34 – 35	105/3184 (3.4%)	54/1556 (3.5%)	51/1628 (3.3%)	0.7366
36 – 37	134/2990 (4.5%)	79/1389 (5.7%)	55/1601 (3.4%)	0.0030
38 – 39	81/2363 (3.5%)	43/1105 (3.9%)	38/1258 (3.2%)	0.3484
40 – 49	81/2298 (3.5%)	41/1173 (3.4%)	40/1125 (3.6%)	0.8491
Overall	947/20363 (4.7%)	597/11048 (5.4%)	350/9315 (3.8%)	< 0.0001

The mean (SD) age was 33.2  ±  5.9 for the 11048 primiparous women and 34.7  ±  5.2 for the 9315 multiparous women (*P*  <  0.0001).

## Discussion

This study demonstrated that a history of prior birth had some favorable effect on reduction in the number of women susceptible to rubella, although this effect was limited, and did not prevent the occurrence of CRS.

As Japanese guidelines for obstetric practice recommend determination of rubella immunity during the 1^st^ trimester with HI test [2], a considerable number of multiparous women with seronegative results (HI titer <  8×) may have realized that they were susceptible to rubella in their previous pregnancies. In addition, as the guidelines recommend postnatal vaccination in women with seronegative results and low HI titer (≤ 16×) [2], a very low frequency of susceptible multiparous women was expected in this study. However, the susceptible fraction decreased only by 30% (from 5.4% to 3.8%) in this study (Table 2). These observations indicated that some women ignored the recommendation and or some obstetricians forgot to recommend the postpartum vaccination. Furthermore, there were no significant differences in seronegative rates between primiparous and multiparous women in some areas. This suggested that the strength of vaccination campaigns for postpartum women with seronegative results differed between areas. A low postpartum vaccination rate of 11% among eligible women has also been reported in other countries [6]. Thus, it was evident based on this study that some women ignore or underestimate the risk of rubella infection during subsequent pregnancies even after the recognition of susceptibility to rubella.

The higher seronegative rate among younger pregnant women is a cause for concern (Figure 2). Although some fluctuations in seronegative rate according to year were seen mainly due to the small size of the study population, seronegative rate was consistently high among teenage pregnant women ranging from 13.0% in 2011 to 23.5% in 2012 and among young women aged 20 – 24 years ranging from 10.7% in 2010 to 14.3% in 2009. Those women may become pregnant in future. According to the serosurvey conducted by the Japanese National Institute of Infectious Diseases (JNIID) in the 5-year period between 2008 and 2012, seronegative rate among females according to age are as follows: 6.7% for women aged 15 – 19 years, 5.5% for those aged 20 – 24, 4.0% for those aged 25 – 29, 3.6% for those aged 30 – 34, 3.2% (41/1299) for those aged 35 – 39, and 3.9% (64/1655) for those aged ≥  40 [7]. Although there was a large discrepancy in seronegative rate between pregnant women in this study and female participants in the serosurvey, especially in younger women, trends such as the higher seronegative rate in younger women were similar between the results of the JNIID serosurvery and our observations. In addition, one in five male Japanese adults in their 30s and 40s was susceptible to rubella [3,7]. The large discrepancy in fraction size susceptible to rubella between younger pregnant women and younger female participants in the serosurvey by the JNIID may be explained as follows: the participants in the serosurvey by the JNIID may have had a greater interest in healthcare than in the general population and teenage pregnant Japanese women may have rather constituted a group at risk for non-vaccination. These speculations were based on the findings that the risk of no antenatal care was high among women with teenage pregnancies in Japan [8], suggesting that women becoming pregnant as teenagers may have been less likely to receive social support from the community. The actual percentage of women susceptible to rubella may have fallen to a figure intermediate between those of pregnant women and female participants in the serosurvey. All of these observations suggested that the current Japanese vaccination strategy has been ineffective for elimination of CRS.

The current Japanese vaccination strategy is ineffective for elimination of CRS for several reasons as follows. With continuing circulation of rubella virus, there is a persistent risk of infection in susceptible pregnant women, even when only 2% – 3% of pregnant women are non-immune [9]. The circulation of rubella virus can occur in the presence of a low vaccination coverage rate in some populations in the community [10-12], as was confirmed in the current outbreak in Japan. The principal rationale for an accelerated vaccination strategy is to reduce the time needed to interrupt rubella virus circulation and to prevent CRS [13]. Eradication of only one manifestation (such as CRS) of a prevalent rubella infection is not a realistic goal. Samuel and John [14] stated that “To eliminate CRS, virus transmission should be interrupted.” Our data combined with those obtained in the serosurvey and the current outbreak strongly suggest that more intensified universal vaccination programs targeting adolescents and children are required and that supplementary immunization activity should be focused on male adults to interrupt endemic rubella transmission. Programs to eliminate rubella have indeed been successful in the USA [15] and appear to have been successful in some European countries [16] and the Americas [12]. It is necessary to realize that “Treatment of CRS is costly and rubella vaccination programs are highly cost-effective” [17].

Japanese guidelines for obstetric practice recommend taking diagnostic measures in women with HI titer ≥  256× during early pregnancy [2]. The total number of women with HI titer ≥  256× was 14.1% (2876/20363) in this study, consistent with the results of a previous study conducted in the 3-year period between July 2003 and June 2006 [18] in which 469 (17.1%) of 2741 women had HI titers ≥  256×. In that study, 411 of the 469 women underwent determination of rubella-specific IgM antibody, 6 women exhibit a positive IgM test result, and none gave birth to a CRS infant [18]. As there are approximately 1.05 to 1.1 million annual births in Japan, these results suggested that the number of women who should undergo determination of rubella-specific IgM would be approximately 150000 yearly in Japan. However, only one infant contracted CRS each year from 2000 to 2003 and 10 infants contracted CRS in the previous rubella outbreak in 2004 [1]. Although several infants may be diagnosed early as having CRS through the diagnostic measures using rubella-specific IgM for pregnant women with HI titers ≥  256×, this strategy for the early detection of CRS may not be cost-effective.

## Conclusion

The experience of prior birth may have favorably affected the reduction in number of pregnant women susceptible to rubella. Only 4.7% (947/20363) of all pregnant women were susceptible to rubella. However, 20 infants with CRS were born during the 12-month period between October 2012 and September 2013 in Japan [3]. Younger women less than 25 years old are more susceptible to rubella than other women of more advanced age. There is still a large fraction of male adults susceptible to rubella in Japan. We may have another rubella outbreak in the near future unless a new vaccination strategy is implemented for the elimination of rubella in Japan.

## Competing interests

All authors declare that they have no financial relationships with biotechnology manufacturers, pharmaceutical companies, or other commercial entities with an interest in the subject matter or materials discussed in the manuscript.

## Authors’ contributions

TY and HM performed statistical analysis, data interpretation and wrote the paper. TY, JM, MH, EH, AO, MI, AN, SS, NU and SM collected and analyzed the data. All authors read and approved the final manuscript.

## Pre-publication history

The pre-publication history for this paper can be accessed here:

http://www.biomedcentral.com/1471-2334/14/152/prepub
